# Dataset on the first weather radar campaign over Lima, Peru

**DOI:** 10.1016/j.dib.2021.106937

**Published:** 2021-03-03

**Authors:** Jairo M. Valdivia, Danny E. Scipión, Marco Milla, Josep J. Prado, Juan C. Espinoza, Darwin Cordova, Miguel Saavedra, Elver Villalobos, Stephany Callañaupa, Yamina Silva

**Affiliations:** Instituto Geofísico del Perú, Peru

**Keywords:** X-band radar, High-resolution, 3D CAPPI, Complex topography, Andes data

## Abstract

The first weather radar campaign over Lima, the capital of Peru, a desertic area on the western side of the Peruvian Andes, was carried out to study the occurrence of rain events in summer 2018. The weather radar was installed strategically and was able to overlook three river basins: Rimac, Chillón, and Lurin. An X-band radar (PX-1000) was used, which operates at 9.55 GHz. PX-1000 was built by the Advanced Radar Research Center (ARRC) at the University of Oklahoma (U.S.A.). The radar operated from January 26th to April 1st, 2018, at Cerro Suche located 2910 m ASL and 55 km from the city of Lima. The PX-1000 performed plan-position-indicators (PPI) for elevations starting at 0° up to 20°. The data presented here were obtained using a three-dimensional constant-altitude plan-position-indicator (3D CAPPI), which was generated by high resolution (250 m) nearest point algorithm.

## Specifications Table

**Table:** 

Subject	Atmospheric Research
Specific subject area	Meteorology
Type of data	Numerical matrix (NetCDF) Table
How data were acquired	X-band polarimetric radar observations
Data format	Raw, Filtered
Parameters for data collection	The data were collected at 11 min and 3 min intervals
Description of data collection	High-spatio-temporal-resolution data based in nearest point algorithm
Data source location	Suche mountain, Santitago de Tuna district Huarochirí province, Lima Perú Latitude and longitude: 11°57′49.7″S 76°32′33.7″W
Data accessibility	First weather radar campaign over Lima, Peru https://doi.org/10.5281/zenodo.4244722 https://scah.igp.gob.pe/sites/datos/PX1000/

## Value of the Data

• It is the first weather radar observations over Lima and surrounding basins, a desertic zone influenced by the complex topography of the Andes.
• These data can be used by scientists and the academic community interested in studying rainfall over desert areas and the topographic effects of the Andes on precipitation.
• This data is useful to evaluate the performance of high-resolution atmospheric models, especially over complex topography.
• The data provide several polarimetric parameters. These polarimetric parameters allow to study the microphysical processes and the impact of microphysics parametrization in the model simulations [1].

## 1 Data Description

This dataset was obtained using the PX-1000, a compact, transportable, and dual polarization X-band weather radar [2]. The PX-1000 characteristics are shown in Table 1. PX-1000 was built by the Advanced Radar Research Center (ARRC) at the University of Oklahoma (U.S.A.), and for the campaign the radar was installed in Santiago de Tuna district (11°57′49.7″S, 76°32′33.7″W), Huarochirí province, in a mountain called “Cerro Suche” at 2910 m ASL and 55 km from the metropolitan city of Lima, the capital of Peru. The PX-1000 was strategically installed to overlook three river basins: Rimac, Chillón, and Lurin. The area is characterized by been arid hot desert near to the coast and arid hot steppe towards to Andes, according to Köppen–Geiger classification [3]. The radar operated from January 26th to April 1st, 2018. The dataset is presented in NetCDF format. The data structure is shown in Table 2. The time in this dataset is in the Matlab format (i.e., number of days since 01–01–0000 UTC). The spatial dimensions are in geographic coordinates, longitude (lon), latitude (lat), and altitude (alt) in Km above sea level. The radar variables in this dataset are: the radar reflectivity factor (Z), radial velocity (V), spectral width (W), differential reflectivity $Z_{dr}$ (D), differential phase shift $\Phi_{dp}$ (P), and co-polar correlation coefficient $\rho_{hv}$ (R). Derivation, meaning and more information on radar variables can be found in [4,5].

**Table 1:** System characteristics of the PX-1000.

**General**	
Operating Frequency	9550 MHz
Typical PRF	2000 Hz
Typical observation range	60 km
**Antenna (Seavey Antenna C082–820)**	
Antenna gain	38.5 dBi
Diameter	1.2 m
3-dB beamwidth	1.8°
Polarimetric isolation	26 dB
Polarization	Dual linear
**Pedestal (Orbit Technology Group AL-4016)**	
Elevation coverage	−2° to 182°
Maximun payload	120 kg
Maximun angular velocity	50° s^−1^
Pointing precision	0.25°
Angular feedback precision	16 bit
**Solid state Transmitters (In-house assembly)**	
Peak power	100 W
Maximun pulse width	69 μs
Typical / maximun duty cycle	4% / 20%
**IF Transceiver (Pentek 7140)**	
IF frequency	50 MHz
Analog-to-digital quantization	14 bit
Receive bandwidth	5 MHz
Typical gate spacing	30 m
Maximum data throughput	320 Mbps
**Experiment configuration**	
Observation range	62.4 km
Range resolution	120 m
Elevation coverage	0° to 20°
Number of sweep elevations	11

*Note:* More PX-1000 technical specification can be found in [2].

**Table 2:** Total rainfall NetCDF structure. (view from the terminal and ncdump tool).

> ncdump -h G3D-20,180,220–204,102.nc netcdf G3D-20,180,220–204,102 { dimensions: time = UNLIMITED; // (1 currently) lon = 481; lat = 481; alt = 33; variables: double time(time); time:long_name = "Days since 01.01.0000 00:00 UTC (MatLab format)"; time:units = "days"; float lon(lon); lon:long_name = "Longitude"; lon:units = "deg"; float lat(lat); lat:long_name = "Latitude"; lat:units = "deg"; float alt(alt); alt:long_name = "Altitude"; alt:units = "Km above sea level"; float Z(alt, lat, lon); Z:long_name = "Radar Reflectivity Factor Z"; Z:units = "dBZ";
float V(alt, lat, lon); V:long_name = "Radial Velocity"; V:units = "m/s"; float W(alt, lat, lon); W:long_name = "Spectral Width"; W:units = "m/s"; float D(alt, lat, lon); D:long_name = "Differencial Reflectivity ZDR"; D:units = "dB"; float P(alt, lat, lon); P:long_name = "Differencial Phase Shift PhiDP"; P:units = "deg"; float R(alt, lat, lon); R:long_name = "Copolar Correlation Coefficient RhoHV"; R:units = "-"; }

## 2 Experimental Design, Materials and Methods

**Fig. 1:**
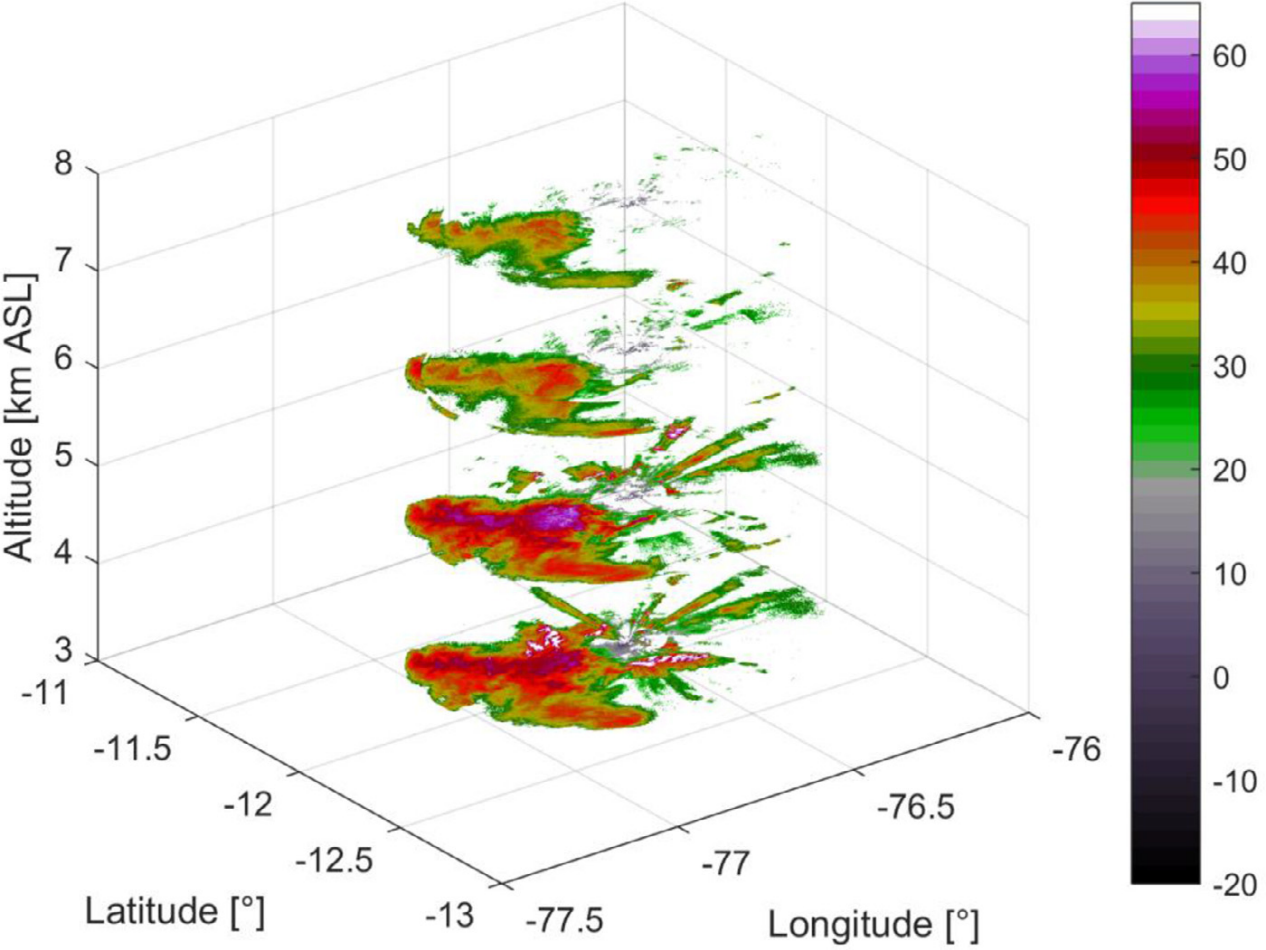
Example of three-dimensional constant-altitude plan-position-indicator (3D CAPPI). This data corresponds to the observations of February 16th, 2018 at 03:01 h. The figure shows four elevations out of thirty-three available.

**Fig. 2:**
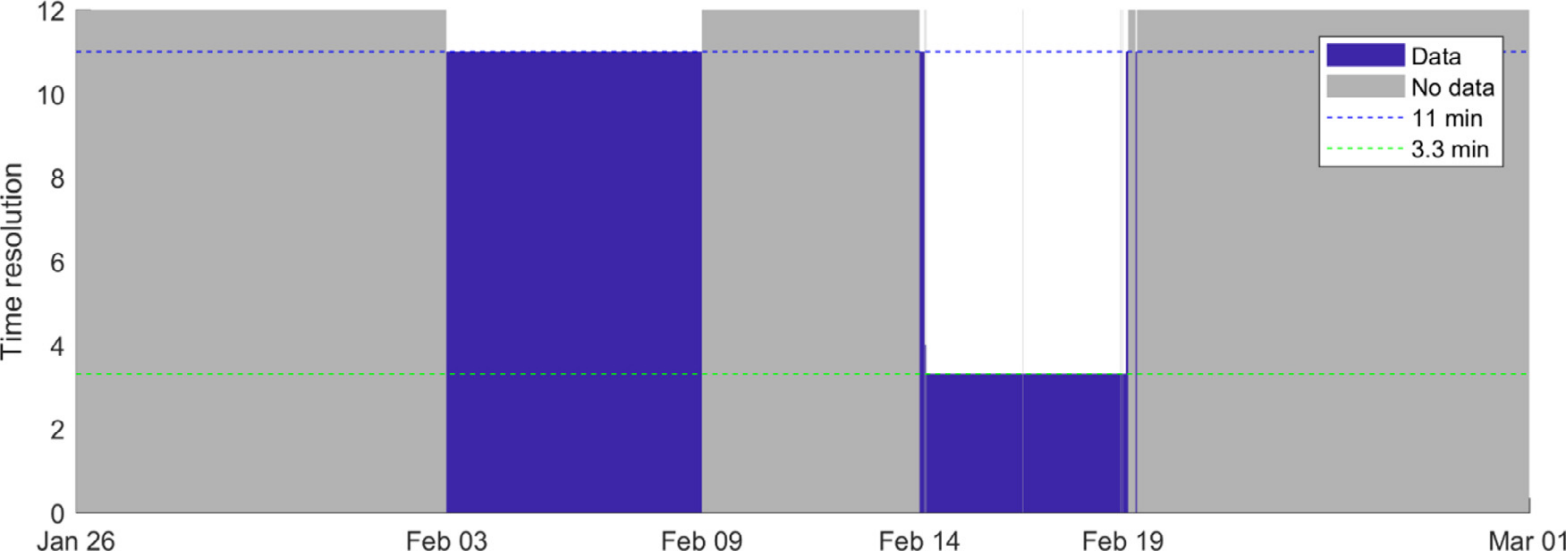
Data availability and time resolution for each period.

This dataset was derived from PX-1000 observational data, which was performed plan-position-indicators (PPI) for elevations starting at 0° up to 20°. A data file was created every time the PPIs from 0° to 20° were completed. The nearest point algorithm was used to create the three-dimensional constant-altitude plan-position-indicator (3D CAPPI) at a resolution of 250 × 250 × 250 m. An example of 3D CAPPI output is shown in Fig. 1. The multiple elevation scan data are not available during the entire campaign period. Due to technical issues, these data are limited to two periods: between February 3rd and 9th the data has a 11 min temporal resolution, and from February 14th to 19th they have 3 min temporal resolution, see Fig. 2.

This dataset become part of the Atmospheric Microphysics And Radiation Laboratory (LAMAR - https://scah.igp.gob.pe/laboratorios/lamar) database, where data is collected from multiple instruments unique to the region. Some data are available open access [6] and interested parties can request other types of data.

## CRediT Author Statement

**Jairo M. Valdivia:** Conceptualization, Data Curation, and Writing - Original Draft; **Danny E. Scipión:** Data curation, Methodology, Writing - Reviewing; **Marco Milla:** Supervision, Writing - Reviewing; **Josep J. Prado:** Data Curation, Validation; **Juan C. Espinoza:** Data Curation, Validation; **Darwin Cordoba:** Data Curation; **Miguel Saavedra:** Data Curation; **Elver Villalobos:** Data Curation; **Stephany Callañaupa:** Data Curation; **Yamina Silva:** Project administration, Funding acquisition, Writing - Reviewing and Editing.

## Declaration of Competing Interest

The authors declare that they have no competing financial interests or other relationships or affiliations that could have appeared to influence the work reported in this paper.
